# Practice explains abolished behavioural adaptation after human dorsal anterior cingulate cortex lesions

**DOI:** 10.1038/srep09721

**Published:** 2015-04-08

**Authors:** H. van Steenbergen, E. Haasnoot, B. R. Bocanegra, E. W. Berretty, B. Hommel

**Affiliations:** 1 Leiden Institute for Brain and Cognition, The Netherlands; 2 Leiden University, Institute of Psychology The Netherlands; 3 Outpatient Clinic for Anxiety Disorders PsyQ, The Hague, The Netherlands

## Abstract

The role of mid-cingulate cortex (MCC), also referred to as dorsal anterior cingulate cortex, in regulating cognitive control is a topic of primary importance in cognitive neuroscience. Although many studies have shown that MCC responds to cognitive demands, lesion studies in humans are inconclusive concerning the causal role of the MCC in the adaptation to these demands. By elegantly combining single-cell recordings with behavioural methods, Sheth et al. [Sheth, S. et al. Human dorsal anterior cingulate cortex neurons mediate ongoing behavioural adaptation. Nature 488, 218–22 (2012).] recently were able to show that neurons in MCC encode cognitive demand. Importantly, this study also claimed that focal lesions of the MCC abolished behavioural adaptation to cognitive demands. Here we show that the absence of post-cingulotomy behavioural adaptation reported in this study may have been due to practice effects. We run a control condition where we tested subjects before and after a dummy treatment, which substituted cingulotomy with a filler task (presentation of a documentary). The results revealed abolished behavioural adaptation following the dummy treatment. Our findings suggest that future work using proper experimental designs is needed to advance the understanding of the causal role of the MCC in behavioural adaptation.

The role of the mid-cingulate cortex (MCC), also referred to as the dorsal anterior cingulate cortex, in regulating cognitive control is a topic of primary importance in cognitive neuroscience. Although many studies have shown that the mid-cingulate cortex responds to cognitive demands^1^, lesion studies in humans are inconclusive concerning the causal role of the MCC in the brain's adaptation to cognitive demands^2^,^3^,^4^. In a recent study by Sheth et al.^5^ (henceforth SHETH), the function of the MCC in cognitive control was investigated using a combination of functional magnetic resonance imaging (fMRI), single-neuronal recoding and behavioural methods, before, and after, precise stereotactic lesioning of MCC in obsessive compulsive disorder (OCD) patients. In line with previous fMRI findings^6^, the authors showed that single neurons in the MCC coded for conflict-induced cognitive demands in a multi-source interference task^7^,^8^ (MSIT) administered before the lesion. Behavioural conflict adaptation^9^,^10^, as indicated by a history-dependent modulation of RT, was observed pre- but not post-lesion. The authors attributed the latter to the cingulotomy, which would support a causal role of the MCC in conflict adaptation.

Considering the wide implications of this innovative experimental finding, we aimed at excluding an alternative theoretical explanation of the diminished post-treatment adaptation effect. In contrast to the robustness of interference effects in conflict tasks such as MSIT and the Stroop task, sequential adaptation effects are known to be transient and may wax and wane depending on certain task parameters^11^,^12^,^13^, including time-on-task^14^. Studies often measure conflict adaptation in a few blocks of trials only, and it has been shown that behavioural adaptation mainly occurs during initial testing and quickly diminishes with practice^14^. Given the nature of SHETH's pre-post experimental design without a control group, it is thus possible that the post-cingulotomy attenuation of conflict adaptation may have been due to practice with the task rather than being an effect of the lesion itself.

In order to test whether time-on-task effects might have played a role in the MSIT task utilized by SHETH, we replicated their design using a control group where subjects were tested before and after a dummy treatment. This dummy treatment substituted cingulotomy of the original study with the presentation of a neutral film. The duration of this filler task matched the time of the original operation. We tested two samples of subjects: a convenience sample of healthy students, and a sample of OCD patients more similar to SHETH's sample. We predicted that if time-on-task indeed reduces adaptation, results should show a reduction of behavioural adaptation in the MSIT task even following a dummy treatment.

## Results

**Figure 1:**
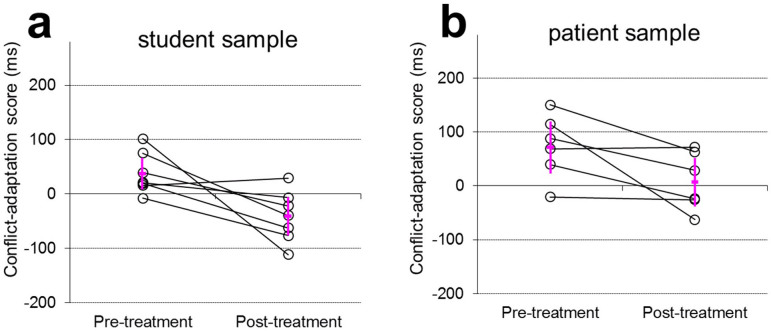
Conflict-adaptation scores in reaction time for individual subjects from the convenience sample (a) and the OCD patient sample (b) before and after the dummy treatment. Error bars indicate means ± 2 standard errors across subjects.

Both samples performed the task accurately (mean error rates 4.9% and 1.6% for the convenience and patient sample, respectively). As Figure 1 shows, the majority of individuals showed reduced behavioural adaptation following the dummy treatment, consistent with the time-on-task hypothesis. Across individuals, statistically reliable pre-treatment adaptation was observed both in the convenience sample, M = 38 ms, t(6) = 2.64, p = 0.039 and in the patient sample, M = 73 ms, t(5) = 3.01, p = 0.030. As expected, no significant post-treatment adaptation was observed for the convenience sample, M = −41 ms, t(6) = −2.34, p = 0.06, and patient sample, M = 9 ms, t(5) = 0.39, p = 0.71. Critically, as predicted, time-on-task significantly reduced adaptation in post-treatment versus pre-treatment scores in both samples: convenience sample: t(6) = 2.93, p_1-sided_ = 0.013; patient sample: t(5) = 2.43, p_1-sided_ = 0.030; samples collapsed: t(12) = 3.94, p_1-sided_ = 0.001. So our control experiment revealed abolished post-treatment adaptation in the absence of a lesion to the MCC. This suggests that time-on-task can significantly influence conflict adaptation in the MSIT task.

## Discussion

Our data suggest that the finding of the diminished post-treatment effect observed by SHETH is open to an alternative theoretical interpretation that does not involve the MCC. Although SHETH convincingly and elegantly demonstrate that MCC neurons encode current and recent conflict, the evidence for a causal role of the MCC in driven behavioural adaptation is still inconclusive^2^,^3^,^4^. Here we showed in a student and patient sample that abolished adaptation in the same MSIT task also occurred following a dummy treatment where subjects simply watched a neutral film fragment that matched the time of the cingulotomy operation of the original study.

An additional issue we want to mention here is that the design of the MSIT task does not properly control for confounds related to feature binding^15^,^16^ and contingency learning^17^. A substantial part of the behavioural adaptation effects observed in SHETH's study and our replication may thus be attributed to simple learning mechanisms rather than adaptations in cognitive control^9^,^12^,^18^. These confounds also preclude the interpretation of the absolute size of adaptation scores in our study. Although our observed pattern of results in the patient group closely parallels the findings by SHETH, the data from our convenience sample was not entirely comparable. Adaptation scores in the patient group were lower than in the patient group and there was a trend for reversed adaptation following the dummy treatment in the former, a finding earlier interpreted in terms of adaptation by binding^19^. Fortunately, very recent studies have shown that confound-free paradigms can be developed that still produce robust behavioural adaptation^20^,^21^,^22^,^23^,^24^. It is recommended that future studies use variants of these tasks to investigate the neural basis of the regulation of cognitive control.

The most compelling and parsimonious explanation for the present and SHETH's findings combined is that time-on-task is sufficient to reduce adaptation effects. This conclusion is also consistent with earlier evidence for time-on-task effects in a different conflict task^14^. Note, however, that we do not want to claim that the MCC does not play a role in behavioural adaptation at all. Indeed, an accumulating body of evidence is showing that the MCC codes cognitive demands and that activity in this area predict subsequent behavioural adaptation^1^,^6^,^25^,^26^,^27^,^28^. The cell recordings by SHETH provide a unique contribution to this ongoing line of research. However, our results conclusively show that alternative experimental set-ups with proper control groups are needed to dissociate practice and lesion effects on behavioural adaptation. Future lesion studies using such designs^2^,^3^,^4^ in combination with confound-free behavioural paradigms will allow us to advance our understanding of the causal role the MCC plays in regulating behaviour.

## Methods

We tested two samples of subjects: a convenience sample of healthy students (mean age: 23; 5 females, 2 males) and a sample of OCD patients (mean age: 42; 4 females, 3 males), which was more similar to the original sample of OCD patients treated with cingulotomy as tested by SHETH. We intentionally used a similarly small sample size as the SHETH study. This allowed us to demonstrate that time-on-task effects can have sufficiently strong effects to account for the effect attributed to cingulotomy as tested in the six subjects described in the SHETH study. The experiment was conducted in accordance with relevant regulations and institutional guidelines and was approved by the local ethics committees from the Faculty of Social and Behavioural Sciences (student sample) and from the Outpatient Clinic for Anxiety Disorders PsyQ (patient sample).

After giving written informed consent, subjects were instructed and performed the same MSIT task^7^,^8^ as described by SHETH. In order to provide a conservative test of the time-on-task hypothesis, we used the smallest number of repetitions in the range of trial repetitions reported by SHETH. That is, we used two blocks of 90 trials instead of the 2–3 blocks (including approximately 100–200 trials each) used by the original study. After a short training block, two blocks of the MSIT task were presented using E-Prime software. These blocks were presented before and after a 23-minute filler task (dummy treatment) where subjects viewed a neutral documentary about house building.

Initial screening of the MSIT behavioural data revealed that one OCD patient produced many errors and omissions in the first two task blocks (10% versus 6%), respectively marked as an extreme value and an outlier in SPSS (> 3.0 IQR versus > 1.5 IQR + 3^rd^ quartile), so this subject was not included in the main analysis (inclusion leads to the same pattern of results with stronger reduction of adaptation t(6) = 2.73, p_1-sided_ = .017). We report MSIT performance in terms of overall error rate and correct reaction time (RT) as a function of current and previous conflict (compatible [C] versus incompatible [I]) trials, after excluding omissions (RT>1500 ms), and post-error trials. As done in earlier work^10^, the standard index of (conflict) adaptation, sensitive to the interaction effect of current and previous conflict, was calculated by subtracting the interference effect following a correct conflict, or incompatible, trial (i) from the interference effect following a correct no-conflict, or compatible, trial (c) (i.e., (cI – cC) – (iI – iC)). Adaptation scores were calculated separately for the pre- and post-treatment task blocks. These scores as well the difference between pre- and post-treatment were submitted to t-tests. We report two-sided p-values unless otherwise noted.

## Author Contributions

H.v.S., E.H., B.B. and E.B. designed the study. H.v.S. and E.H. acquired and analyzed data. H.v.S. and B.B. wrote the main manuscript text and E.H., E.B. and B.H. reviewed the manuscript.
